# Urban Diets Linked to Gut Microbiome and Metabolome Alterations in Children: A Comparative Cross-Sectional Study in Thailand

**DOI:** 10.3389/fmicb.2018.01345

**Published:** 2018-06-22

**Authors:** Juma Kisuse, Orawan La-ongkham, Massalin Nakphaichit, Phatthanaphong Therdtatha, Rie Momoda, Masaru Tanaka, Shinji Fukuda, Siam Popluechai, Kongkiat Kespechara, Kenji Sonomoto, Yuan-Kun Lee, Sunee Nitisinprasert, Jiro Nakayama

**Affiliations:** ^1^Department of Bioscience and Biotechnology, Faculty of Agriculture, Kyushu University, Fukuoka, Japan; ^2^Institute of Food Research and Product Development, Kasetsart University, Bangkok, Thailand; ^3^Specialized Research Unit: Probiotics and Prebiotics for Health, Faculty of Agro-Industry, Kasetsart University, Bangkok, Thailand; ^4^Institute for Advanced Biosciences, Keio University, Tsuruoka, Japan; ^5^Intestinal Microbiota Project, Kanagawa Institute of Industrial Science and Technology, Kawasaki, Japan; ^6^Transborder Medical Research Center, University of Tsukuba, Tsukuba, Japan; ^7^PRESTO, Japan Science and Technology Agency, Saitama, Japan; ^8^School of Science, Mae Fah Luang University, Chiang Rai, Thailand; ^9^Human Gut Microbiome for Health Research Unit, Mae Fah Luang University, Chiang Rai, Thailand; ^10^Sooksatharana Co., Ltd., Bangkok, Thailand; ^11^Department of Microbiology, National University of Singapore, Singapore, Singapore

**Keywords:** gut microbiota, 16S rRNA gene sequencing, fecal metabolomics, short-chain fatty acid, Peptostreptococcaceae, high-fat diet, vegetable-based diet, Thailand

## Abstract

Loss of traditional diets by food globalization may have adverse impact on the health of human being through the alteration of gut microbial ecosystem. To address this notion, we compared the gut microbiota of urban (*n* = 17) and rural (*n* = 28) school-aged children in Thailand in association with their dietary habits. Dietary records indicated that children living in urban Bangkok tended to consume modern high-fat diets, whereas children in rural Buriram tended to consume traditional vegetable-based diets. Sequencing of 16S rRNA genes amplified from stool samples showed that children in Bangkok have less Clostridiales and more Bacteroidales and Selenomonadales compared to children in Buriram and bacterial diversity is significantly less in Bangkok children than in Buriram children. In addition, fecal butyrate and propionate levels decreased in Bangkok children in association with changes in their gut microbial communities. Stool samples of these Thai children were classified into five metabolotypes (MTs) based on their metabolome profiles, each characterized by high concentrations of short and middle chain fatty acids (MT1, *n* = 17), amino acids (MT2, *n* = 7), arginine (MT3, *n* = 6), amino acids, and amines (MT5, *n* = 8), or an overall low level of metabolites (MT4, *n* = 4). MT1 and MT4 mainly consisted of samples from Buriram, and MT2 and MT3 mainly consisted of samples from Bangkok, whereas MT5 contained three samples from Bangkok and five from Buriram samples. According to the profiles of microbiota and diets, MT1 and MT2 are characteristic of children in Buriram and Bangkok, respectively. Predicted metagenomics indicated the underrepresentation in MT2 of eight genes involved in pathways of butyrate biosynthesis, notably including paths from glutamate as well as pyruvate. Taken together, this study shows the benefit of high-vegetable Thai traditional diets on gut microbiota and suggests that high-fat and less-vegetable urban dietary habits alter gut microbial communities in Thai children, which resulted in the reduction of colonic short chain fatty acid fermentation.

## Introduction

The gut microbiota consists of hundreds of microbial species, collectively 100 trillion cells, that play an important role in the interface between food intake and host health. It aids in nutrient metabolism as well as conditioning the gut environment for host health. Notably, some commensals benefit the host by fermenting dietary fibers into short-chain fatty acids (SCFAs) (Morrison and Preston, 2016; Ríos-Covián et al., 2016). SCFAs play pivotal roles in the maintenance of intestinal homeostasis by serving as an energy source for colonic epithelial cells and also as signals for host receptors involved in regulation of immune cell proliferation and lipid and glucose metabolism (Furusawa et al., 2013; Kimura et al., 2013; Canfora et al., 2015). Furthermore, among SCFA, butyrate plays a crucial role as a histone deacetylases (HDAC) inhibitor involved in epigenetic regulation of regulatory T-cell development and maintenance (Smith et al., 2013). An abnormal imbalance in the gut microbiota, called “dysbiosis,” is associated with deprivation of SCFAs in the intestine, resulting in the loss of the above benefits notably leading to a leaky gut and subsequent metabolic endotoxemia (Shen et al., 2013; Llorente and Schnabl, 2015).

Foods are recognized as important to maintaining the microbiota and facilitating efficient production of SCFAs (Flint et al., 2015; Kovatcheva-Datchary et al., 2015; Ríos-Covián et al., 2016). However, modern diets tend to hamper the gut microbiota ecosystem, which may explain the recent increase in the incidence of certain non-communicable diseases such as inflammatory bowel, allergy, and metabolic diseases, as well as colorectal cancer (Ou et al., 2013; West et al., 2015; Statovci et al., 2017). Indeed, African children and Italian children were found to have distinct gut microbial compositions and different concentrations of SCFAs (De Filippo et al., 2010). Further, the change from a native African to an African American diet increased the expression of genes involved in butyrate biosynthesis in intestines of participants and also reduced the levels of their mucosal biomarkers of cancer risk (O’Keefe et al., 2015). In addition, our previous study on the gut microbiota of children in Leyte island in the Philippines indicated that children living in urban and rural cites have distinct microbial compositions that correspond to those in African and Western people (Nakayama et al., 2017).

Diets in Thailand are notably rich in a variety of unique and healthy foods, some of which have had a strong impact on the dietary cultures of surrounding countries. Particularly, the Thai traditional diet has been relatively high in vegetables and low in fat (Kosulwat, 2002). A study that investigated traditional Thai foods showed that 22 recipes were associated with lower rates of carcinogenic mutations (Kangsadalampai and Plaingam, 2008). Furthermore, it was reported that a wide variety of traditional Thai food ingredients such as lemon grass and galangal root have powerful anti-tumor properties (Murakami et al., 1994). However, Thai food culture is changing rapidly, i.e., becoming westernized, with an increase in fat, sugar, and animal protein content (Kosulwat, 2002). Sugar consumption among Thais, for example, increased nearly threefold, from 12.7 to 33.2 kg per person per year, between 1983 and 2006. Some surveys have shown that an increase in fat consumption is associated with an increase in the incidence of heart disease and that high sugar consumption is linked to diabetes (Wibulpolprasert, 2008) One report indicated that the expenditure by Thais on Western-style fast food increased by 40% from 1999 to 2005 (United States Department of Agriculture [USDA], 2009). These data indicate that Western-style food has come to play a significant role in Thai food culture.

The gut microbial communities of Thai people have been reported previously (La-ongkham et al., 2015; Nakayama et al., 2015; Ruengsomwong et al., 2016). Among Asians, Thai people have shown unique distribution pattern of enterotypes, which are a summary measure of the gut microbiota. Enterotypes of Thai children differ by city, whereas children in other countries examined tended to be colonized by one enterotype across cities (Nakayama et al., 2015). In fact, *Prevotella*-types represent the majority of children in Khon Kaen, whereas *Bacteroides*-type represents the majority of children in Bangkok. Furthermore, guts of adult vegetarians were mostly colonized by *Prevotella*-type, whereas those of non-vegetarians were mostly colonized by *Bacteroides*-type (Ruengsomwong et al., 2016). These previous studies suggested that modern less-fiber and high-fat diets are altering the Thai gut microbiota.

**FIGURE 1:**
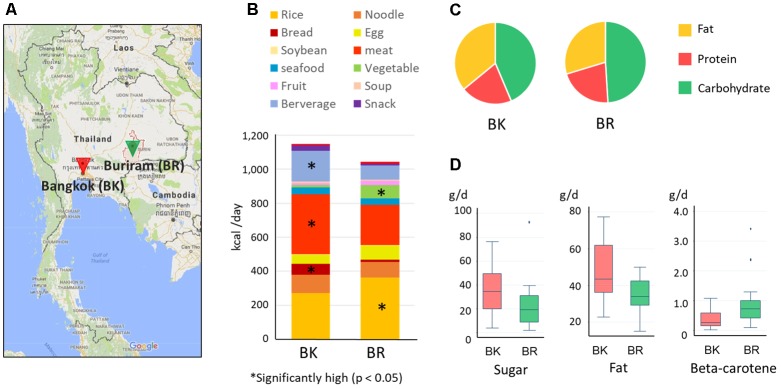
Sampling cities and diets of participants in this study. **(A)** Map of Bangkok and Buriram. The map was generated based on Google Maps 2017 (https://www.google.com/maps/). “BK” and “BR” were used as abbreviations. **(B)** Average daily dietary intake for participants in the two cities. The contribution of each food group was estimated from the 7-day dietary record reported by participants’ parent/guardian and was converted to energy units (kcal) according to databases of food energy and nutrition. Asterisks indicate statistically significant differences (*p* < 0.05) by Wilcoxon rank-sum test. More detailed information on food consumption is shown in Supplementary Tables S1, S2. **(C)** The energy ratio of macronutrients consumed daily in BK and BR children. **(D)** The nutrients in foods consumed daily showing statistically significant differences between BK and BR children (*p* < 0.05 by Wilcoxon rank-sum test). The box plots show the smallest and largest values, 25 and 75% quartiles, medians, and outliers.

Thus far, a number of studies have indicated the impact of Western diets on gut microbiota through the comparative cross-sectional studies across continents (De Filippo et al., 2010; Yatsunenko et al., 2012; Lin et al., 2013; Martínez et al., 2015; Turroni et al., 2016). In addition, some recent studies have shown the transition of gut microbiota as a contrast between urban and rural within country (De Filippo et al., 2017; Nakayama et al., 2017). The present study aims to deepen our insight into the value of traditional local diet and the impact of modern diet on the gut microbiota of young Asians in developing countries in South–East Asia. To this end, we conducted a comparative cross-sectional study in urban and rural cites in Thailand (**Figure 1A**). Among rural cities, we chose Buriram city, located in the Isan region, which is a geographically contiguous area in North–Eastern Thailand. The population is largely Thai and consumes traditional Thai (Isan) diet. Notably, many local Isan dishes are low in fat and high in vegetable and herbal content (Seubsman et al., 2009a). Furthermore, a survey targeting youth in Isan indicated that they show cultural resistance to modern diets (Seubsman et al., 2009b). Bangkok which we chose as urban city is cosmopolitan where residents are affected by urbanized environment, notably dilution of their traditional foods by Western or modern diets. The structure and functionality of their gut microbiota were monitored in terms of fecal microbiome and metabolome configurations.

## Materials and Methods

### Study Design

To estimate the number of subjects satisfied a statistical power in this study, we computed the PERMANOVA power based on the variance and difference of fecal microbiome data obtained in our previous study of Thai children. We used the micropower R-package (Kelly et al., 2015), in which the weighted UniFrac distance matrix was simulated based on within-group distance variance (mean and standard deviation) of the data from 26 Bangkok children and therein a range of effect sizes were generated by incorporating a range of group differences. Using the simulated matrices, the PERMANOVA powers were calculated for varying effect sizes (ω^2^) and sample sizes (Supplementary Figure S1). As a result, 10 subjects per group afford 80% power to detect an ω^2^ of 0.10 which corresponds to the difference between the Bangkok children and children in Khon Kaen, a rural city in Thailand. Ten subjects per group afford 80% power to detect an ω^2^ of 0.05 which corresponds to the effect observed in a controlled-feeding study (Wu et al., 2011) and 15 subjects per group afford 100% power to detect the same effect size. Accordingly, we aimed more than 15 subjects per group. Details of this power analysis are described in Supplementary Methods.

We recruited Thai children who were born and raised in Bangkok or Buriram city, and aged 9–11 years. Subjects who were administered antibiotics for 2 weeks prior to sampling or suffered from diarrhea for 1 month prior to sampling were excluded. Eventually, we used 17 children from Bangkok and 28 children from Buriram for this study. Characteristics of the participants in this study are described in Supplementary Table S1 and summarized in **Table 1**.

**Table 1:** Characteristics of the participants in this study.

	Bangkok (*n* = 17^a^)	Buriram (*n* = 28^b^)	*p*^c^
Age (y)	10.47 ± 0.72	9.79 ± 0.63	<0.05
Gender (male/female)	13/3	12/16	<0.05
Height (cm)	145.4 ± 11.1	139.5 ± 7.4	0.11
Weight (kg)	45.6 ± 12.2	34.4 ± 8.0	<0.05
BMI (kg/m^2^)	21.3 ± 4.0	17.4 ± 2.9	<0.05

*^a^Two samples were missing for dietary records and one sample was missing for gender information. ^b^Two samples were missing for dietary records and three samples were missing for metabolome analysis. ^c^Except for gender, the statistical significance was assessed by the Welch’s t-test. For gender, the statistical significance was assessed by the Fisher’s exact test.*

Fresh stool samples were collected from these 45 children and were subjected to the 16S rRNA gene sequencing and metabolome analysis as described below. The parents/guardians reported 1-week dietary records as described below as well as answered a questionnaire that addressed the children’s physiological characteristics. Welch’s *t*-test was performed using the Excel *t*-test function (Microsoft Excel 2016). As described in the footnotes, some samples and dietary records were missing because of failure by participants. There were statistical differences in age, gender, weight, and body mass index (BMI) between children in Bangkok and Buriram. The confounding effects of age and gender biases were examined as described below.

### Dietary Information

Diets of children participating in this study were recorded by children’s parents/guardians, using a dietary record form that asked about the menu, ingredients, and quantity of every meal, including breakfast, mid-morning snack, lunch, mid-afternoon snack, dinner, and after-dinner snack for 7 days before stool samples were collected. Using the INMUCAL-Nutrients V3 database NB1 program (Institute of Nutrition, Mahidol University, 2013), recorded foods were categorized and their energy (kcal) and nutrient contents (g, mg, or μg) were estimated. The energy from each food group and its fat portion were calculated per day per person and subjected to Wilcoxon rank-sum test in R ver. 3.3.2 to determine the statistical significance of differences between children in Bangkok and Buriram (Supplementary Tables S2-1, S2-2, respectively). Each nutrient group consumed by an individual was also calculated per day and subjected to the Wilcoxon rank-sum test to determine the statistical significance of differences between children in Bangkok and Buriram (Supplementary Table S2–3).

### Stool Sample Collection and Processing

Fresh feces were collected into a 76 × 20-mm sterile container with 2 mL of RNAlater (Ambion, Inc., Austin, TX, United States) for microbial genomic extraction and a 55 × 44-mm sterile container for metabolite extraction (Sarstedt, Nümbrecht, Germany). The samples were placed on ice immediately for transfer to a -20°C freezer. For the metabolome analysis, the fecal samples were lyophilized and then stored at -80°C. Details of the stool sample collection and storage are described in Supplementary Methods.

### 16S rRNA Gene Sequencing

Bacterial genomic DNA was extracted from stool samples as described previously (Nakayama et al., 2015) (For details, see Supplementary Methods). The variable region, V1–V2, of the 16S rRNA gene was amplified from the fecal genomic DNA (1 ng) using TaKaRa Ex Taq HS (Takara Bio, Shiga, Japan) and universal primers, Tru 27F (5′-CGC TCT TCC GAT CTC TGA GRG TTT GAT YMT GGC TCA G-3′) and Tru 354R (5′-TGC TCT TCC GAT CTG ACC TGC CTC CCG TAG GAG T-3′). The amplified products were then used as templates for a second PCR for further amplification with barcode-tag primers. The second-PCR products were purified using a QIAquick PCR Purification Kit (Qiagen, Valencia, CA, United States) according to the manufacturer’s protocol. The amplified DNA was quantified using a PicoGreen dsDNA Assay Kit (Life Technologies, Eugene, OR, United States) per the manufacturer’s protocol. All DNA samples were then mixed in an equal amount and purified by electrophoresis in a 2% (wt/vol) agarose gel, followed by extraction from the gel using a FastGene Gel/PCR Extraction Kit (Nippon Genetics Co., Ltd., Tokyo, Japan). The purified DNA was applied to paired-end sequencing using a Illumina MiSeq v3 chemistry (Illumina Inc., San Diego, CA, United States).

### Processing of 16S rRNA Gene Sequences

The obtained sequences were processed using the Uparse pipeline in Usearch v9.2^1^ (Edgar, 2013). The pairs of raw sequence reads were merged using the fastq_mergepairs script with mismatch windows up to 25 bases. High quality sequences were selected from the merged sequences using the fastq_filter script with an expected error score lower than 1.0 and then PCR chimera-like sequences were removed by employing the Uchime algorithm. The high-quality sequences obtained were clustered using the cluster_otus script, resulting in 663 non-singleton operational taxonomic units (OTUs). The taxonomy of each OTU was assigned using the SINTAX command (Edgar, 2016) with RDP training set v16 and a cut-off value of 0.8. The raw merged sequences before quality filtering were mapped to OTUs with identities higher than 0.97, using the usearch_global script, and the number of reads per sample assigned to each OTU was counted. Eventually, 11020 ± 3948 reads per sample were assigned. The OTU table was subsampled for a sequence depth equal to 5,000 for all samples for the following analysis (The OTU table is deposited in Supplementary Table S3-1). The values of good’s coverage for the samples were 99.23 ± 0.18% (minimum = 98.83%), indicating sufficient sequencing depth for the microbiome investigation in this study.

To test the statistical significance of differences in the fecal bacterial community structure between Bangkok and Buriram children, pairwise weighted UniFrac distance was calculated by using the beta_diversity.py command in QIIME version 1.9.1^2^ (Kuczynski et al., 2012), and was subjected to PERMANOVA test using the PERMANOVA function in the micropower R-package (Kelly et al., 2015). The taxonomic composition of each sample was determined, using the summarize_taxa_througy_plots.py command in QIIME version 1.9.1^2^ (Kuczynski et al., 2012) (The data is deposited in Supplementary Tables S3-2–4). To determine the statistical significance of differences in the abundance of each bacterial group in samples from Bangkok and Buriram, the Wilcoxon rank-sum test was performed. To determine the statistical significance of differences between the five metabolotype groups, a pairwise Wilcoxon rank-sum test was performed in R ver. 3.3.2. To examine the confounding effects of age and gender, we performed the multivariate regression analysis in Stata SE12.0 (Stata Corporation, College Station, TX, United States), for the taxonomy composition using age and city, or gender and city as the independent variables.

### Principal Component Analysis (PCA)

Principal component analysis was performed using the rda function in the Vegan package of R ver. 3.3.2. Principal component 1 (PC1) and 2 (PC2) were subjected to the Wilcoxon rank-sum test to determine the statistical significance of differences between samples from Bangkok and Buriram.

### Alpha Diversity Analysis

The number of observed OTUs, and ACE and Chao1 alpha diversity indices were determined at a sequence depth of 5,000 reads per sample with 10 random iterations, using the QIIME alpha_rarefaction.py script. The statistical differences of these indices between Bangkok and Buriram were examined by the Wilcoxon rank-sum test. To determine the statistical significance of differences in the Chao1 index between the five metabolotype groups, the pairwise Wilcoxon rank-sum test was performed.

### Linear Discriminant Analysis Effect Size (LEfSe)

The LEfSe (Segata et al., 2011) was calculated using the online version of Galaxy^3^. For OTUs with an average abundance in all samples that was greater than 0.1%, abundances were normalized to the sum of the values per sample in 1 million and then subjected to linear discriminant analysis (LDA). The LDA was performed using a one-against-all strategy, and OTUs showing a score higher than 2.0 were selected.

### Phylogenetic Investigation of Communities by Reconstruction of Unobserved States (PICRUSt)

Representative OTU sequences were subjected to a Basic Local Alignment Search Tool (BLAST) analysis using the Greengenes reference sequence database (gg_13_5) (DeSantis et al., 2006). The OTU table annotated according to the reference sequence was applied to a PICRUSt analysis online^4^, version 1.0.0 (Langille et al., 2013). The OTU table was normalized by the 16S rRNA copy number per chromosome, and the number of KEGG genes in each sample was counted using a KEGG gene content table [ko_13_5]. The relative abundance of each KEGG gene in each sample was calculated as the number of KEGG genes divided by the total number of genes. Further, each KEGG gene was annotated to KEGG pathway and the relative abundance of each KEGG pathway group was estimated for each sample. These abundances of KEGG genes and KEGG pathways in each sample were subjected to the one-way ANOVA test and Welch’s *t*-test to determine the significance of differences among or between metabolotype groups, respectively. KEGG genes determined significantly abundant in a certain group were mapped using the KEGG Mapper Search Pathway tool^4^.

### Capillary Electrophoresis Time of Flight Mass Spectrometry (CE-TOF MS) Measurement

Metabolites in the stool samples were analyzed as previously described (Soga et al., 2003; Mishima et al., 2017). Briefly, fecal metabolites were extracted by vigorous shaking with methanol containing 20 μM each of methionine sulfone, D-camphol-10-sulfonic acid, and 2-(*N*-morpholino) ethanesulfonic acid as the internal standards, and then cleaned with chloroform and water extraction and ultrafiltration using Ultrafree-MC (UFC3 LCC NB, 5,000 NMWL, black label). The purified fractions were analyzed using a CE-TOF MS system consisting of an Agilent CE capillary electrophoresis system (Agilent Technology, Palo Alto, CA, United States), Agilent G32500AA LC/MSD TOF system (Agilent Technologies), Agilent 1100 series binary high-performance liquid chromatography pump, G1603A Agilent CE-MS adapter, and G1607A Agilent CE-ESI-MS sprayer kit. For the analysis, metabolites were quantified using a standard curve obtained from standard samples. Concentrations below the detection limit were substituted with zero, and metabolites whose levels were below the detection limit in all of the samples were excluded. The data of CE-TOF MS is deposited in Supplementary Table S4.

Pearson’s correlation between samples was calculated using whole metabolite composition and then subjected to hierarchical clustering. A heat map was created using the heatmap.2 function in the ggplots2 package of R ver. 3.3.2.^5^ To find metabolites abundant or depleted in a specific metabolotype group, the concentration of each metabolite was subjected to Wilcoxon rank-sum test between one group and all other groups combined (see the list in Supplementary Table S4). To determine the statistical significance of differences in the amounts of certain metabolites in the metabolotype groups, a pairwise Wilcoxon rank-sum test was performed with or without Bonferroni adjustment in R ver. 3.3.2. Pearson’s correlation between each metabolite and bacterial abundance was determined by Pearson’s correlation coefficient test in Stata SE12. To examine the confounding effects of age and gender, we performed the multivariate regression analysis in Stata SE12 using butyrate or propionate concentration as dependent variable and age and city, or gender and city as the independent variables.

### *Post Hoc* Power Analysis

A *post hoc* power analysis was conducted with G^∗^Power software 3.1.9.2 (Faul et al., 2007) to retrospectively examine the observed power in the Wilcoxon rank-sum test and the Pearson’s correlation analysis. The Power level (1-β error probability) was calculated by the two-tailed *t*-test with the number of used sample at the significance level (*p* = 0.05) and the effect size level determined by the mean and standard deviation for the Wilcoxon rank-sum test or the *R^2^* for the Pearsons’ correlation analysis.

### Accession Number of the 16S rRNA Gene Sequences

The raw sequence data were deposited in the DNA Data Bank of Japan (DDBJ) sequence read archive (DRA006238) under BioProject no. PRJDB5860, which contains links and access to the stool sampling data (BioSample SAMD00097316 to SAMD00097371).

## Results

### Differences in Dietary Habits Between Children in Bangkok and Buriram

Based on the 7-day dietary records, we summarized the daily diets of children in Bangkok and Buriram as shown in **Figures 1B–D**) (see Supplementary Table S2-1 for details). Buriram children consumed more rice and vegetables, whereas children in Bangkok consumed more bread, meat, and beverages. Notably, consumption of vegetables in Bangkok children was considerably reduced corresponding to 1.0% of total calorie intake, whereas Buriram children consumed them almost every day corresponding to 7.3% of total calorie intake. All of the participating children in both cities consumed rice, particularly steamed rice, almost every day, suggesting that rice was the main carbohydrate source for Thai children. However, its frequency and amount consumed were much greater in Buriram children (10.2 times per week, 207.1 kcal/day) than in Bangkok children (6.3 times per week, 140.8 kcal/day). Furthermore, Buriram children consumed more glutinous rice (101.4 kcal/day), served in the traditional Isan style, than Bangkok children (13.1 kcal/day). On the other hand, Bangkok children consumed more single-dish rice (94.4 kcal/day), such as fried rice (see Supplementary Table S2-1), than Buriram children (40.3 kcal/day). Total calorie intake was slightly higher in Bangkok children, although the difference between their intake and that of children in Buriram was not statistically significant.

We then estimated quantities of each nutrient consumed in individuals using the food-and-nutrient database in Thailand. A PCA using the log-ratio transformed nutrient matrix data showed a significant differences of dietary habits between children in Bangkok and Buriram (*p* < 0.001 in PERMANOVA analysis, Supplementary Figure S2). Indeed, children in Bangkok consumed significantly more fat and sugar and less beta-carotene than children in Buriram (**Figures 1C,D** and Supplementary Table S2-3). The fat consumption ratio among Bangkok children was statistically higher than among Buriram children (35.9 ± 5.9% vs. 29.7 ± 5.3%, *p* = 0.0017 in Student’s *t*-test). The statistical power for this comparison was 0.92 at *p* = 0.05 in the *post hoc* analysis, indicating sufficient power of this study to address the difference in the dietary habit between these two groups. The difference in the fat consumption ratio was a result of the higher intake of fried rice, cooked breads, and processed meats by Bangkok children (Supplementary Table S2-2) and the difference in the intake of beta-carotene reflects a lower rate of vegetable consumption in Bangkok children than in Buriram children (Supplementary Table S2-1). In summary, dietary habits of participating children in Bangkok were significantly modernized and westernized, whereas Buriram children had more traditional Thai dietary habits.

### Difference in Fecal Bacterial Communities Between Children in Bangkok and Buriram

**FIGURE 2:**
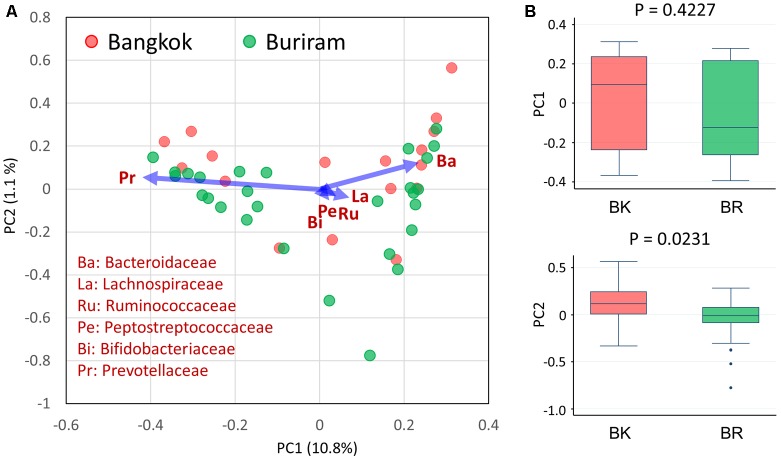
Principal component analysis (PCA) of fecal bacterial community profiles (family level) of children in Bangkok and Buriram. **(A)** PCA plot of 17 Bangkok and 28 Buriram children. **(B)** PC1 (upper) and PC2 (lower) sample distributions. The probability value (*p*) was determined by Wilcoxon rank-sum test. Refer to **Figure 1D** for a description of the box plot.

We analyzed the bacterial composition of stool samples by amplicon sequencing of the 16S rRNA V1–V2 region. Then, we profiled the bacterial community structures of 45 samples using pairwise weighted UniFrac distances. As unanticipated, there was no statistical differences in the community structures between Bangkok and Buriram groups, even though the statistical power reached adequate level to detect a large and middle effect size such as enterotype differentiation or impact by controlled-diet feeding (Kelly et al., 2015). Therefore, we profiled the bacterial community at family level by the PCA (**Figure 2A**). Three enterotype-like variations were observed, although they were not entirely discrete: the first one, localized in the PC1-positive region, was defined by a high abundance of Bacteroidaceae; the second one, localized in the PC1-negative region, was defined by a high abundance of Prevotellaceae; and the third one, localized in the PC2-negative region, was defined by a high abundance of Bifidobacteriaceae, Lachnospiraceae, Ruminococcaceae, and Peptostreptococcaceae. Although there was no obvious relationship between the city of residence and the enterotype-like cluster, Bangkok and Buriram samples tended to be driven in PC2-positive and -negative directions, respectively (**Figure 2B**).

**FIGURE 3:**
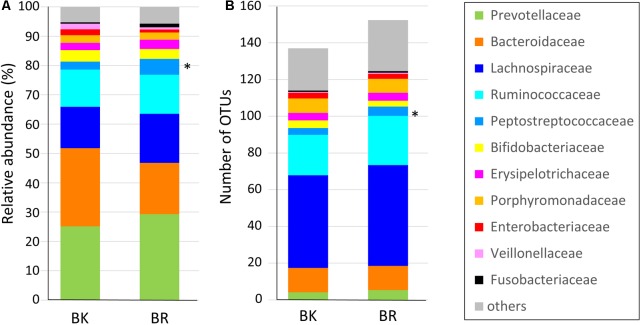
Composition of the fecal microbiota of children in Bangkok and Buriram. **(A)** Relative abundance of bacterial families. Average abundance of bacteria in 17 Bangkok (BK) and 28 Buriram (BR) children. **(B)** The average number of operational taxonomic units annotated to each family in 17 BK and 28 BR children. The asterisk at the right side of the bar indicates the bacterial family that was significantly more abundant in BR children than BK children (*p* < 0.05, Wilcoxon rank-sum test).

**FIGURE 4:**
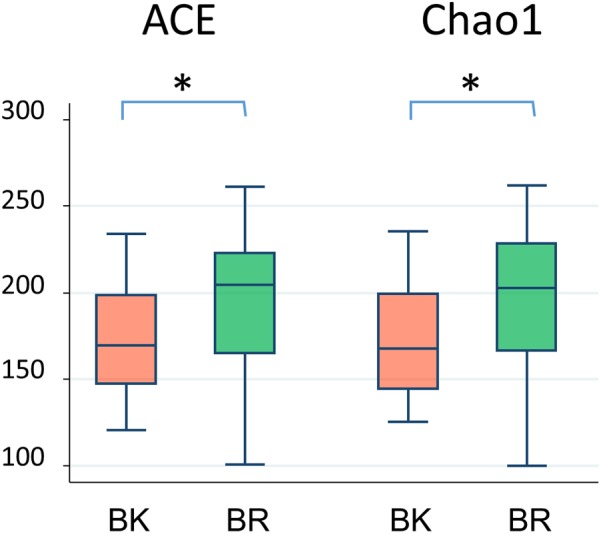
Alpha-diversity of fecal microbiota of children in BK and BR. ACE and Chao1 indices were statistically compared between Bangkok and Buriram samples by the Wilcoxon rank-sum test. Asterisks indicate *p* < 0.05.

**Figure 3** shows the family level composition of fecal microbiota by city. There was a tendency for Bacteroidaceae to be less abundant in Buriram children, whereas some Clostridia families such as Lachnospiraceae and Peptostreptococcaceae were more abundant in Buriram children. **Figure 3B** shows the number of OTUs belonging to each family. More than a half of over 100 total OTUs were found to belong to class Clostridia, especially in the families Lachnospiraceae and Ruminococcaceae, whereas two dominant families, Prevotellaceae and Bacteroidaceae, consisted of less than 20 OTUs. A subdominant family, Peptostreptococcaceae, was statistically more abundant and more diverse in Buriram samples than in Bangkok samples, as shown in **Figures 3A,B**, respectively. Total alpha diversity tended to be higher in Buriram samples than in Bangkok samples, as indicated by Chao1 (*p* = 0.042) and ACE (*p* = 0.047) species richness estimates (**Figure 4**) and the number of total OTUs shown in **Figure 3B** (*p* = 0.07).

**FIGURE 5:**
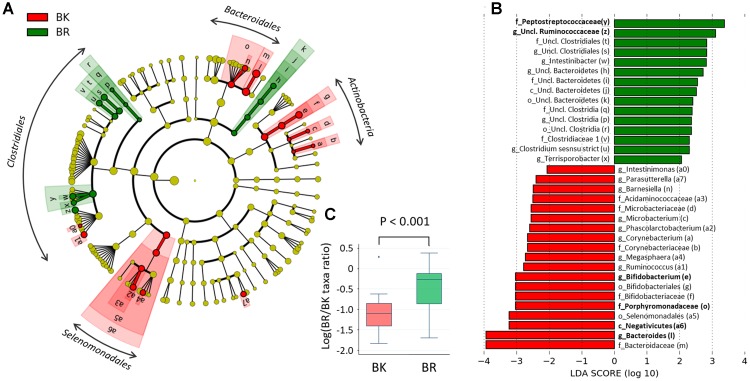
Linear discriminant analysis effect size (LEfSe) analysis to identify differences in abundant taxa between Bangkok and Buriram samples. **(A)** Cladogram showing different abundant taxa between samples from Bangkok and Buriram (LDA score > 2.0, *p* < 0.1). Alphabets correspond to those in parentheses in **(B)**. **(B)** Taxonomic groups showing LDA scores > 2.0 with *p* < 0.1. p, phylum; c, class; o, order; f, family; g, genus. **(C)** Boxplot showing the distribution of the log-ratios of the BR/BK taxa ratio. The significance of difference between two cities was examined by the Wilcoxon rank-sum test. Effect size and power of this statistical analysis were calculated to be 1.01 and 0.88, respectively, by *post hoc* analysis. Refer to **Figure 1D** for a description of the box plot.

To find more detailed differences in the fecal bacterial compositions between children in the two cities, a LEfSe analysis was performed using phylum to genus-level data. As shown in **Figure 5A**, Bangkok children were more highly colonized by classes Actinobacteria and orders Selenomadales and Bacteroidales, whereas Buriram children were more highly colonized by some taxonomic groups belonging to the order Clostridiales, such as families Peptostreptococcaceae and unclassified Ruminococcaceae. In **Figure 5B**, the relative abundance of taxonomic groups showing an LDA score greater than 10^3^ (taxa indicated by bold letters in **Figure 5B**) was summed for the Bangkok type (Negativicutes + *Bacteroides*+ Porphyromonadaceae + *Bifidobacterium*) and the Buriram type (Peptostreptococcaceae + unclassified Ruminococcaceae), and the ratio of the sum of the Buriram type to the sum of the Bangkok type was calculated for each child (henceforth, the ratio is called the BR/BK taxa ratio). The BR/BK taxa ratio was significantly different between children in Bangkok and Buriram, as shown in **Figure 5C** (*p* < 0.001 in Wilcoxon rank-sum test). The statistical power for this comparison was 0.88 at *p* = 0.05 in the *post hoc* analysis, indicating sufficient power of this study to address the alteration in the gut microbial community between Bangkok and Buriram children.

To examine the confounding effects of age and gender biases on city of residence, we performed a multivariate regression analysis of the BR/BK taxa ratio. The results indicated that the city of residence was strongly associated with the BR/BK taxa ratio even after the adjustment, suggesting there was no confounding effect by age or gender in the analysis of gut microbiota community structure (Supplementary Tables S5A,B).

### Difference in Concentrations of SCFAs in Stool Samples Between Children in Bangkok and Buriram

**FIGURE 6:**
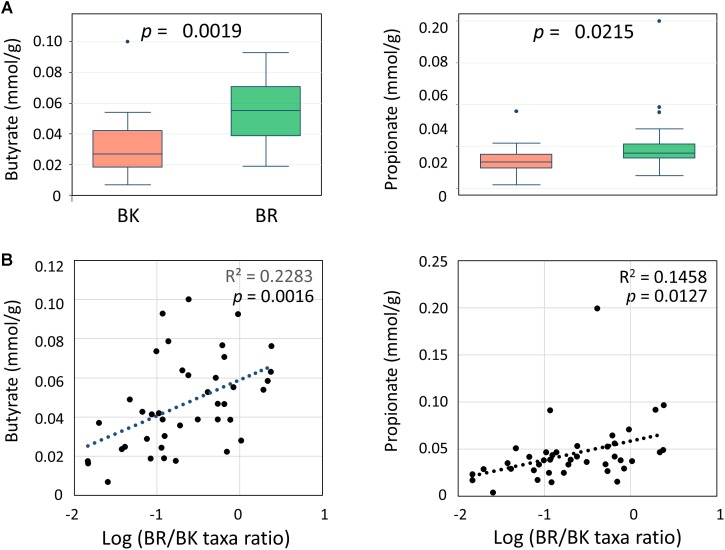
Comparison of the concentrations of fecal SCFAs between children in Bangkok and Buriram **(A)** and correlations with gut bacterial composition **(B)**. The probability value (*p*) was calculated by Wilcoxon rank-sum test. Refer to **Figure 1D** for a description of the box plot. Correlation statistics were determined by Pearson’s correlation test. The *post hoc* analysis indicated the statistical powers for these Pearson’s correlation analyses to be 0.94 for butyrate and 0.82 for propionate.

We performed a CE-TOF MS-based fecal metabolome analysis and found that butyrate and propionate concentrations differed significantly between children from the two cities (**Figure 6A**). Further, butyrate concentrations positively correlated with BR/BK taxa ratios with sufficient statistical significance and power (**Figure 6B**). To examine the confounding effect of age and gender on city of residence, we performed a multivariate regression analysis of the butyrate and propionate levels. The results indicated that the city of residence strongly associated with both the butyrate level and the propionate level even after the adjustment, suggesting there was no confounding effect by age and gender in the analysis of SCFA levels (Supplementary Tables S5C–F).

### Five Metabolotypes Found in the Fecal Metabolite Profiles of Children in Bangkok and Buriram

**FIGURE 7:**
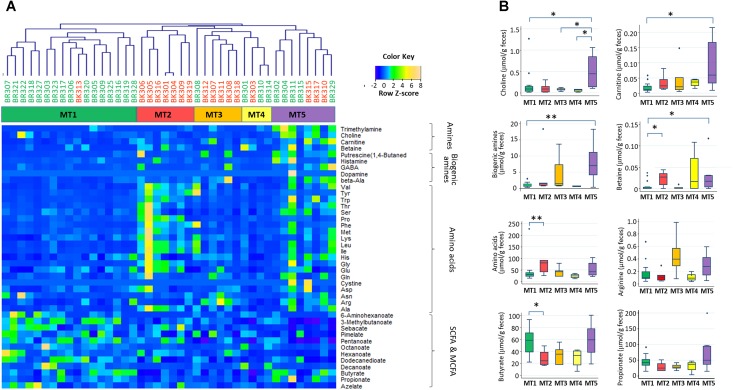
Five metabolotypes based on the measurement of 214 metabolites in stool samples from children in Bangkok and Buriram. Metabolites characterizing fecal metabolotypes were selected, and their abundances are shown in a heat map **(A)** and box plots **(B)**. **(A)** A hierarchical clustering dendrogram was generated based on Pearson’s correlation determined based on the abundance of whole metabolites (see Supplementary Figure S3). Samples colored by subjects’ city of residence and clusters colored by metabolotype were shown between the dendrogram and heat map. The abundance of each metabolite was converted to a *Z*-score across all samples and displayed in the heat map according to the above color key. **(B)** Single and double asterisks indicate *p* < 0.1 and *p* < 0.05, respectively, in a pairwise Wilcoxon rank-sum test with Bonferroni adjustment. “Biogenic amines” represent the sum of beta-alanine, dopamine, gamma-aminobutyric acid, histamine, phenethylamine, putrescine, and tyramine. “Amino acids” represent the sum of 20 standard proteinogenic amino acids. Refer to **Figure 1D** for a description of the box plot.

**FIGURE 8:**
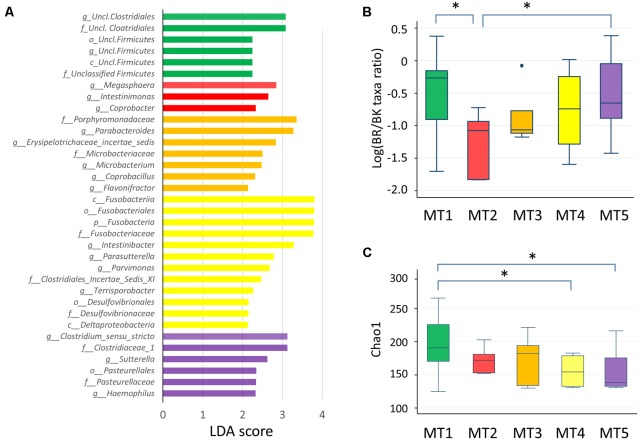
Fecal microbiota of five metabolotype groups. **(A)** Taxa overrepresented in each metabolotype group. Taxa with linear discriminant analysis scores >2.0 with *p* < 0.05 are shown. p, phylum; c, class; o, order; f, family; g, genus. **(B)** The BR/BK taxa ratio in each metabolotype group. **(C)** Chao1 index scores in each metabolotype group. Asterisks indicate *p* < 0.05 in a pairwise Wilcoxon rank-sum test without Bonferroni adjustment. Refer to **Figure 1D** for a description of the box plot.

Using fecal metabolome data, a cluster analysis was performed, with five clusters resulting (**Figure 7**, see Supplementary Figure S3 for whole metabolites). These clusters were characterized by high abundance of SCFAs in metabolotype-1 (MT1), amino acids in metabolotype-2 (MT2), arginine in metabolotype-3 (MT3), and amines in metabolotype-5 (MT5), whereas metabolotype-4 (MT4) totally lacked in metabolites (see Supplementary Table S4 for details). The bacterial compositions of these five metabolotype groups were compared by LEfSe analysis (**Figure 8A**).

Except for one sample from a child in Bangkok, all of the MT1 samples were derived from Buriram children. MT1 samples contained middle chain fatty acids (MCFAs) such as capric and caproic acids, in addition to two SCFAs measured. The microbiota of MT1 samples was characterized by a high BR/BK taxa ratio and high alpha diversity (**Figures 8B,C**), as observed in Buriram children. On the other hand, MT2 samples were characterized by high concentrations of proteinogenic amino acids (**Figure 7**). All of the MT2 samples were derived from Bangkok children and showed a low BR/BK taxa ratio (**Figure 8B**). MT3 was also mainly derived from Bangkok children. The bacterial composition of this group was characterized by a high abundance of *Parabacteroides*, whereas the BR/BK taxa ratio was intermediate between those of MT1 and MT2. Taken together, MT1 represents the high-SCFA-producing microbiota of Buriram children, and MT2 represents the low-SCFA producing microbiota of Bangkok children.

MT4 was characterized by the overall low level of fecal metabolites. This group comprised three samples from Buriram and one from Bangkok. The microbiota of MT4 was characterized by a high abundance of *Fusobacterium* and Desulfovibrionaceae and low alpha diversity (**Figure 8C**).

MT5 comprised five Buriram and three Bangkok samples. This type was characterized by high concentrations of choline, betaine, and carnitine, which are generally abundant in red meat and dairy products, and also a series of biogenic amines such as gamma-aminobutyric acid (GABA) and putrescine. A number of previous studies have shown that these meat-derived amines are converted to trimethylamine by gut bacteria and further metabolized by a liver enzyme to trimethylamine *N*-oxide, which is associated with metabolic syndrome, fatty liver disease, and cancer (Velasquez et al., 2016). In the stool samples from Thai children, trimethylamine concentrations were positively correlated with amine levels and were significantly more abundant in participants in the MT5 group (Supplementary Figure S4). The microbiota associated with MT5 samples was characterized by a high abundance of the genera *Haemophilus* and *Sutterella* (**Figure 8A**) and low alpha diversity (**Figure 8C**).

### Predicted Metagenomics Showing Differences in the Metabolism Between MT1 and MT2

**FIGURE 9:**
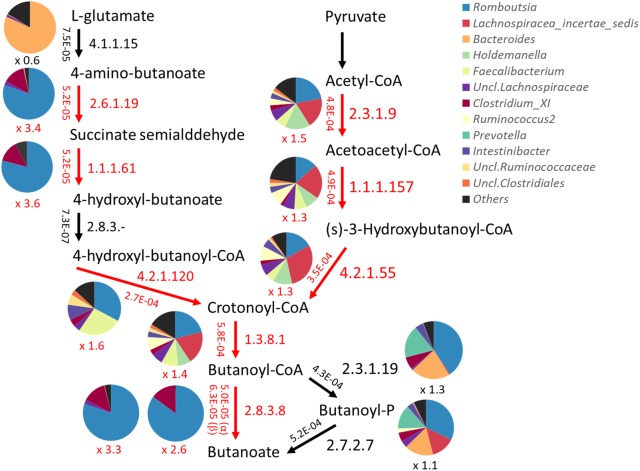
Predicted abundance of genes involved in the butyrate biosynthetic pathway. Abundances of genes were predicted by PICRUSt and are shown beside the arrow in each path represented by the EC number. Pie charts represent the contribution of bacteria genera to each path. Red arrows and letters indicate paths significantly overrepresented in Buriram samples.

Genes and their functions encoded by the fecal bacterial community were quantitatively predicted by PICRUSt based on 16S rRNA gene profiles. The abundance of each KEGG pathway group was compared among the metabolotypes. As a result, KEGG pathways involved in “metabolism” and some other subcategories were shown to be overrepresented in MT2 group compared with other groups, particularly MT1 (Supplementary Figure S5). Therefore, we compared the abundances of all KEGG annotated genes between groups MT1 and MT2, and genes overrepresented in the MT1 group were mapped in the KEGG pathway (Supplementary Table S6). The results showed that MT1 was enriched in genes involved in the metabolism of plant-related compounds, such as flavonoid, carotenoid and limonene. MT2 was enriched in nitrogen metabolism including amino acid metabolism, as well as lipid and energy metabolism, whereas MT1 was enriched in ketone body metabolism and phosphotransferase system as well as butanoate metabolism. Notably, 8 genes were overrepresented in butyrate biosynthesis pathways in MT1, including paths from glutamate as well as pyruvate (**Figure 9**). Those genes involved in the paths from glutamate are mainly encoded by bacteria in the genus *Romboutsia*, family Peptostreptococcaceae, whereas those in the paths from pyruvate are encoded by various genera, although *Romboutsia* is one of major contributors. Further, 19 genes involved in phosphotransferase system, including cellobiose phosphotransferase, were overrepresented in MT1, suggesting that higher capacity of MT1 microbiome to ferment a variety of sugars. There was no statistical difference in the total abundance of propanoate metabolism between MT1 and MT2. However, 8 genes, including propionate CoA-transferase [EC:2.8.3.1] gene, were statistically enriched in the pathway of propanote metabolism of MT1 group (data not shown).

### Link of Dietary Nutrients to Gut Microbiome and Metabolome

**FIGURE 10:**
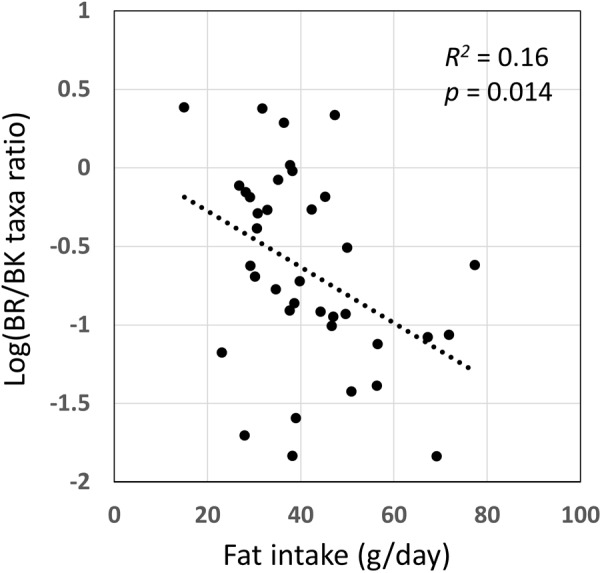
Correlation of fat intake level with BR/BK taxa ratio. Pearson’s correlation analysis was performed between fat intake level and the BR/BK taxa ratio and a significant correlation (*p* < 0.05) was obtained. The *post hoc* analysis indicated the statistical powers for this Pearson’s correlation analysis to be 0.85.

**FIGURE 11:**
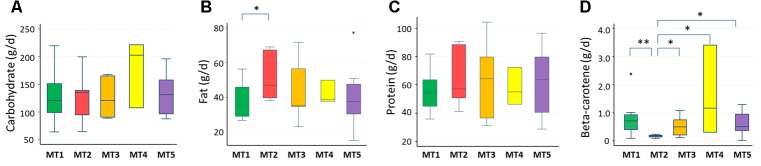
Levels of nutrients consumed by children in each metabolotype group. **(A)** Carbohydrates, **(B)** fats, **(C)** proteins, **(D)** beta-carotene. Double and single asterisks indicate *p* < 0.05 in pairwise Wilcoxon rank-sum tests with and without Bonferroni adjustment, respectively. Refer to **Figure 1D** for a description of the box plot.

We examined the correlation between dietary nutrients and gut microbiome in Thai children. As a result, fat intake level showed a significant negative correlation with the BR/BK taxa ratio as shown in **Figure 10** (*R^2^* = 0.16, *p* = 0.014 by Pearson’s correlation analysis, and Power = 0.85 in *post hoc* analysis), suggesting the significant impact of high level fat consumption on the gut microbiota. Furthermore, we examined the association between dietary nutrient and fecal metabolotypes (**Figure 11**). Among the macronutrients, only fat consumption showed a significant association with metabolotypes. In addition, beta-carotene intake level significantly differed among the metabolotypes groups. Children in MT2 group consumed more fat and much less beta-carotene than those in other metabolotype groups, especially MT1. Altogether, the differences in dietary habits and gut environment between two cities were reflected to the two metabolotypes, MT1 and MT2.

### Correlation of Metabolotype With Body Mass Index

**FIGURE 12:**
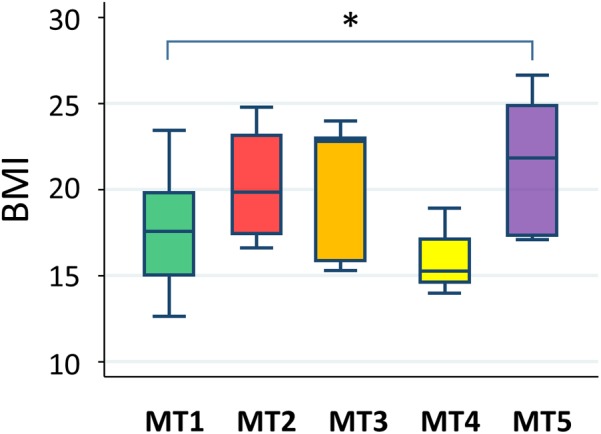
Body mass index level in each metabolotype group. The asterisk indicates *p* < 0.05 in a pairwise Wilcoxon rank-sum test without Bonferroni adjustment. Refer to **Figure 1D** for a description of the box plot.

A statistically significant difference was only found between groups MT1 and MT5 (**Figure 12**). According to data from the National Health Examination Surveys and the National Health and Nutrition Examination in the United States, the BMI (kg/m^2^) of 9-year-olds with normal or healthy weight ranged from 14.0 to 18.6 and, in 11-year-olds, ranged from 14.5 to 20.2. The median BMIs in the MT2, MT3, and MT5 groups were over 20, suggesting that children in these groups tended to be overweight, whereas participants in group MT1 were mostly categorized as having normal or healthy weights. Particularly, three out of eight children in group MT5 were categorized as obese.

## Discussion

To investigate the influence of urbanization of dietary habit on the gut microbial community structure and their function, we conducted this comparative cross-sectional study with a relatively small but statistically reasonable sample size. As a result, we found a signature of urbanization in the intestinal microbiome and metabolome configurations of Bangkok children. In fact, participants from these two cities showed distinct dietary habits: Buriram children more or less retained Isan traditional dietary habits, which include foods low in fat and high in vegetables, whereas Bangkok children consumed more sugar and fat, perhaps reflecting the urbanization of their dietary culture. The average total fat intake in Bangkok children was more than 35% of total energy intake, the upper level recommended for children by the Food and Agriculture Organization (FAO, 2010). The dietary records in this study indicate that the higher amount of fat intake in Bangkok children depends on cooking style more than raw materials: for example, many fried meals were served to Bangkok children. The consumption of less vegetables by children in Bangkok, particularly those with an MT2 metabolotype, was remarkable. This is reflected in the low consumptions of beta-carotene.

In our previous study on the gut microbiota in an Asian population, two enterotype-like clusters, one driven by *Prevotella* and the other one driven by *Bacteroides*, were found, and overrepresentation of each enterotype was observed in distinct populations, such as *Prevotella*-type microbiota in Thai vegetarians (Ruengsomwong et al., 2016), and in the rural cities of Khon Kaen in Isan region of Thailand (Nakayama et al., 2015), and Baybay on Leyte island in the Philippines (Nakayama et al., 2017). These results suggested that low-fat and vegetable-based diets promote the colonization by *Prevotella*-type microbiota. The *Prevotella*/*Bacteroides* trade-off was also found in a study comparing the gut microbiota of children from Burkina Faso consuming a rural African diet rich in dietary fiber and Italian children consuming a modern Western diet (De Filippo et al., 2010). Further, recent studies have observed the *Prevotella/Bacteroides* trade-off within a country (De Filippo et al., 2017; Nakayama et al., 2017), suggesting that the enterotype shift is ongoing in developing countries. Unexpectedly, however, we did not observe the enterotype shift between Bangkok and Buriram children. Preliminary data showed that the enterotypes were not consistent in this Thai cohort across samplings with a 1-month interval, while most Japanese children were consistently colonized by a *Bacteroides*-type enterotype. This could suggest that the gut microbiota of Thai children is now being influenced by modern diets and are in a state of transition.

Rather than the enterotypes reflected in PC1 of the PCA, the city of residence correlated somewhat with PC2, which mainly reflected the Firmicutes-to-Bacteroidetes (F/B) ratio. The F/B ratio is known to be positively correlated with high-fat diet-induced obesity (Ley et al., 2006; Turnbaugh et al., 2008, 2009; Hildebrandt et al., 2009; Zhang et al., 2012). In this study, however, Bangkok children, who tended to consume a high-fat diet and to be overweight, showed a lower F/B ratio. This agrees with a line of studies suggesting that species, community, or functional level studies are required to understand the causation between diets and microbiota (Duncan et al., 2008; Schwiertz et al., 2010; Daniel et al., 2014). Indeed, some families or genera, such as *Bacteroides*, *Bifidobacterium*, and *Porphyromonadaceae*, are commonly higher in children in Italy (De Filippo et al., 2017) as well as children in Bangkok, suggesting a niche established in the gut environment of urban children.

A number of studies have demonstrated that plant-based diets promote colonic fermentation of SCFAs and have a profound effect on host health (David et al., 2014; Simpson and Campbell, 2015; Turroni et al., 2016). A line of African children gut microbiota studies have commonly indicated the high level of the series of SCFAs (De Filippo et al., 2010, 2017). Although acetate was not measurable in our CE-TOF-MS system, the rural children in Thailand also showed higher level of propionate and butyrate than Bangkok children. The line of studies on Africans and Asians demonstrated that their rural microbiomes have higher capacity to accept a variety of sugars, including overrepresentations of phosphotransferase genes and carbohydrate-active enzyme genes (Rampelli et al., 2015). These results suggested that human gut microbiome primarily evolved to utilize a wide range of carbon source and was then narrowed down under the excessive intakes of simple sugars.

The cluster analysis based on the top–down metabolomics data generated MT1 representative of Buriram metabolome. Samples in the MT1 group were characterized by higher concentrations of SCFAs and higher alpha diversity. It is interesting that the MT1 samples also showed higher concentrations of MCFAs. Microbial production of MCFAs is not common in the human gastrointestinal tract. They may be because of the consumption of coconut milk or palm oil, which are common ingredients in Thai food. However, we could not find any correlation between MCFAs in stool samples and the dietary records of MT1 children.

MT2, characterized by a high abundance of amino acids and low level of SCFAs, showed a lower BR/BK taxa ratio representative of the microbiota in Bangkok children. The dietary habits of MT2 children were characterized by high-fat and remarkably low-vegetable consumption. Although there is no direct evidence, this suggests that their urbanized dietary habit altered the gut microbial communities of Bangkok children and reduced the microbial production of SCFAs. Whereas SCFAs in the intestine benefit the host, overrepresentation of amino acids in the gut has been found to associated with gut dysbiosis in patients with Crohn’s disease (CD) (Ni et al., 2017). This paper reported an ^15^N flux study in mice that showed bacterial urease releases ammonia by hydrolysis of host urea and allows the transfer of host-derived nitrogen to the gut microbiota as a source of amino acid biosynthesis.

Interestingly, the predicted metagenomics showed that Peptostreptococcaceae, which was overrepresented in Buriram children, was involved in the biosynthesis pathway from glutamate to butyrate. Although Peptostreptococcaceae is not a common butyrate producer such as *Faecalibacterium* or *Roseburia* (Louis and Flint, 2009), there are some reports showing that this taxonomic group can produce SCFAs from amino acids (Chen and Russell, 1988; Wilson et al., 2000; Dai et al., 2011). The path from amino acids to butyrate may play an important role in maximizing SCFA production from limited carbon sources in the colon.

MT4, characterized by the overall low level of fecal metabolites, had a high abundance of *Fusobacterium* and Desulfovibrionaceae, which are known to be overrepresented in patients with colon cancer and ulcerative colitis and to be involved in inflammation (Ohkusa et al., 2003; Rowan et al., 2010; Castellarin et al., 2012; Figliuolo et al., 2017). It is notable that the BMIs of these MT4 subjects were lower than those of the other groups, except for those in the MT1 group, which might be related to their unusual gut microbiota and environments. On the other hand, MT5 was characterized as rich in metabolites, especially amines and amino acids. It is interesting that the CD patients in the aforementioned paper were also abundant in fecal amines, similar to patients with inflammatory bowel disease reported elsewhere (Ni et al., 2017; Santoru et al., 2017). Furthermore, a high abundance of *Haemophilus* belonging to potentially pathogenic bacteria in MT5 subjects was also common in CD patients (Ni et al., 2017). Although numerous studies have shown, in terms of physiology as well as toxicology, the diverse activity of biogenic amines, those in the intestine should be further investigated. It should be noted that the same contrast in fecal amino acids and biogenic amines was observed in Italians and Hadza hunter-gatherers (Turroni et al., 2016), suggesting that dysbiosis-like metabolomic changes are a sign of stress in gut microbial communities under the urbanization of diets.

## Conclusion

This study suggests that the transition of dietary habit from traditional to modern distorted the functionality of gut microbiota of Thai children, notably SCFA productivity, through alteration of their community structure. As a number of previous studies have demonstrated that plant-based diets promote colonic fermentation of SCFAs and have a profound effect on human health, the high-vegetable Thai traditional diets appears to benefit children in Thailand and the lower level of fecal SCFAs appears to be a risk marker of dietary urbanization in Thailand. A larger scale community-based cohort study is warranted to monitor the impact of urban diets on the health and disease of youngster in developing countries in South–East Asia.

## Ethics Statement

This study was approved by the ethics committee of the Faculty of Agriculture of Kyushu University under approval no. 13-005 and jointly approved by the ethics committees of the Bangkok Hospital Phuket under ethic approval no. sq. 001/2016. Written informed consent was obtained from the parents/guardians of all participants. We entered and analyzed all samples and questionnaire data anonymously and published them using participant identification numbers.

## Author Contributions

JK, OL-o, MN, SF, KS, Y-KL, SP, SN, and JN conceived and designed the experiment. OL-o, MN, SP, KK, and SN provided the sample collection. JK, OL-o, MN, PT, RM, MT, SF, and JN performed the experiment. JK, OL-o, MN, SF, and JN analyzed the data. JK, OL-o, MN, PT, SF, Y-KL, SN, and JN wrote the paper.

## Conflict of Interest Statement

The authors declare that the research was conducted in the absence of any commercial or financial relationships that could be construed as a potential conflict of interest.

## Footnotes

1 http://drive5.com/usearch/manual/upp_ill_pe.html

2 http://qiime.org/

3 http://huttenhower.sph.harvard.edu/galaxy/root, version 1.0.0

4 http://www.genome.jp/kegg/tool/map_pathway1.html, version 1.0.0

5 https://cran.r-project.org/web/packages/gplots/gplots.pdf#search=%27heatmap.2%27
