# Supplementary material for: Urban Diets Linked to Gut Microbiome and Metabolome Alterations in Children: A Comparative Cross-Sectional Study in Thailand
Source: Front Microbiol. 2018 Jun 22;9:1345. doi: 10.3389/fmicb.2018.01345 (PMC6024022; doi:10.3389/fmicb.2018.01345)
Supplement: Supplementary file 7 [file Data_Sheet_1.DOCX]

**Supplementary Methods**

**Urban Diets Linked to Gut Microbiome and Metabolome Alterations in Children: A Comparative Cross-sectional Study in Thailand.**

*Juma Kisuse, Orawan La-ongkham, Massalin Nakphaichit, Phatthanaphong Therdtatha, Rie Momoda, Massaru Tanaka, Shinji Fukuda, Siam Popluechai, Kongkiat Kespechara, Kenji Sonomoto, Yuan-Kun Lee, Sunee Nitisinprasert, Jiro Nakayama*

**Stool sample collection.**

Participants collected two stool samples, for 16S rRNA gene analysis and metabolome analysis, respectively, from fresh feces defecated on the trail paper. For 16S rRNA gene analysis, three spatula portions (total 0.3 ~ 0.5 g) were collected into a sterile tube (AS ONE Corporation, Osaka, Japan) containing 2 mL of RNA later (QIAGEN K.K., Tokyo, Japan). For metabolome analysis, one big spatula portion (> 1 g) were collected into a sterile container (55 x 44 mm, Sarstedt). Both were immediately placed on pre-frozen ice pack in a styrofoam box and transferred to the laboratory within 6 h. The samples for 16S rRNA gene analysis were stored in −20 °C freezer until DNA extraction. The stool samples for metabolome were tentatively stored at −80 °C freezer and lyophilized, and then stored at −80 °C freezer until metabolite extraction.

**DNA extraction**

Bacterial DNA was extracted from samples by the bead-beating method and purified as described previously (1), with some modification. In brief, stool sample was diluted 10-fold with RNAlater and homogenized. Then, 200 µl of the fecal sample diluents were mixed with 1 ml PBS and vortexed. After centrifugation at 20,000 × g for 5 min at 4 ºC, the supernatant was removed and washed twice with 1 ml of PBS buffer to remove PCR inhibitors. The supernatant was discarded and the pellet was stored at –30 ºC until use. Three hundred milligram of glass beads (diameter, 0.1 mm) (TOMY SEIKO Co., Ltd., Tokyo, Japan), 300 μl of Tris-SDS solution and 500 μl of TE buffer-saturated phenol were added to a thawed sample, and then vortexed vigorously using a FastPrep FP120 (Bio 101) at a speed of 5.0 m/sec for 30 s. Four hundred microliter of phenol/chloroform/isoamyl alcohol (25:24:1; v/v) was added to 400 μl of supernatant and shook vigorously with the use of FastPrep PF120 at a speed of 4.0 m/sec for 45 s. After centrifugation at 20,000 × g for 5 min at 4 ºC, 250 μl of supernatant was mixed with 25 μl of 3 M sodium acetate (pH 5.2). After being kept for 3 min on ice, 300 μl of ice cold 100% isopropanol was added and centrifuged at 20,000 × g for 5 min at 4 ºC. The pellet of DNA was washed in 500 μl of ice cold 70% ethanol and air dried prior to suspension in 1 ml of TE buffer (pH 8.0) and stored at –30 ºC until use.

Reference

1. Matsuki T, Watanabe K, Fujimoto J, Takada T, Tanaka R. Use of 16S rRNA gene-targeted group-specific primers for real-time PCR analysis of predominant bacteria in human feces. Appl Environ Microbiol 2004; 70: 7220-7228.

**Power and sample-size estimation**

The OTU composition data of 52 Thai children containing 26 Bangkok and 26 Khon Kaen children (2) was used as a reference data to estimate power and sample-size estimation.

Simulation of the PERMANOVA power was performed in the “micropower” R-package (3). The variance and standard deviation of pairwise weighted Unifrac distance among the 26 samples was calculated using ‘dist_mean_sd_var” function. As a result, the variance and standard deviation were 0.256 and 0.069, respectively. To test the significance of difference of the bacterial community between Bangkok and Buriram children, the OTU composition data containing the 52 children data was applied to the PERMANOVA analysis in the micropower R-package with 1000 bootstrap permutation.

The *p*, *R^2^*, and ω^2^ values were determined to be 0.000999, 0.121, and 0.102, respectively.

Then, we simulated a set of matrices of pairwise distances for which within-group distances match the variance of distances of 0.256. In order to determine the level of subsampling and number of OTUs necessary to model the expected within-group distances, we applied the ‘hashMean’ and ‘hashSD’ functions, simulating 100 OTUs and subsampling to retain 9% of OTUs generated the desired mean within-group distance of 0.256 and within-group distance standard deviation of 0.069. A set of OTU tables, incorporating a range of between-group effects in addition to the desired within-group-distance distribution, was then generated using the ‘simPower’ function. Having calculated pairwise distances for each simulated OTU table, we proceeded with power analysis using the bootPower function and assessed the PERMANOVA power with the varied number of subjects per group.

Reference

2. Nakayama J, Watanabe K, Jiang J, Matsuda K, Chao S-H, Haryono P, La-Ongkham O, Sarwoko M-A, Sujaya IN, Zhao L, et al. Diversity in gut bacterial community of school-age children in Asia. *Sci Rep* (2015) **5**:8397. doi:10.1038/srep08397

3. Kelly BJ, Gross R, Bittinger K, Sherrill-Mix S, Lewis JD, Collman RG, Bushman FD, Li H. Power and sample-size estimation for microbiome studies using pairwise distances and PERMANOVA. *Bioinformatics* (2015) **31**:2461–8. doi:10.1093/bioinformatics/btv183
