# Supplementary figures and images for: Urban Diets Linked to Gut Microbiome and Metabolome Alterations in Children: A Comparative Cross-Sectional Study in Thailand
Source: Front Microbiol. 2018 Jun 22;9:1345. doi: 10.3389/fmicb.2018.01345 (PMC6024022; doi:10.3389/fmicb.2018.01345)

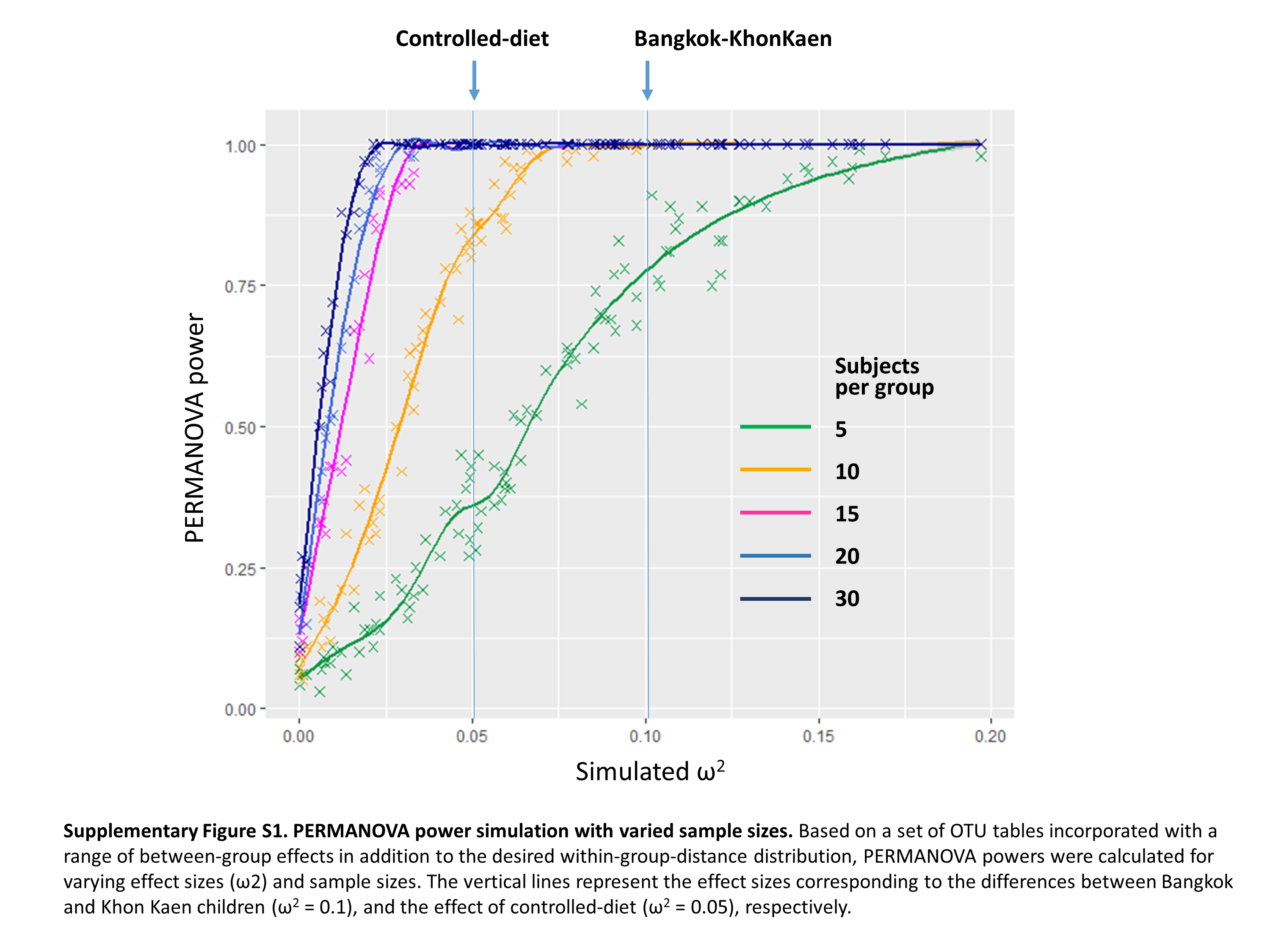

Supplement: Supplementary file 8 [file Image_1.TIF]

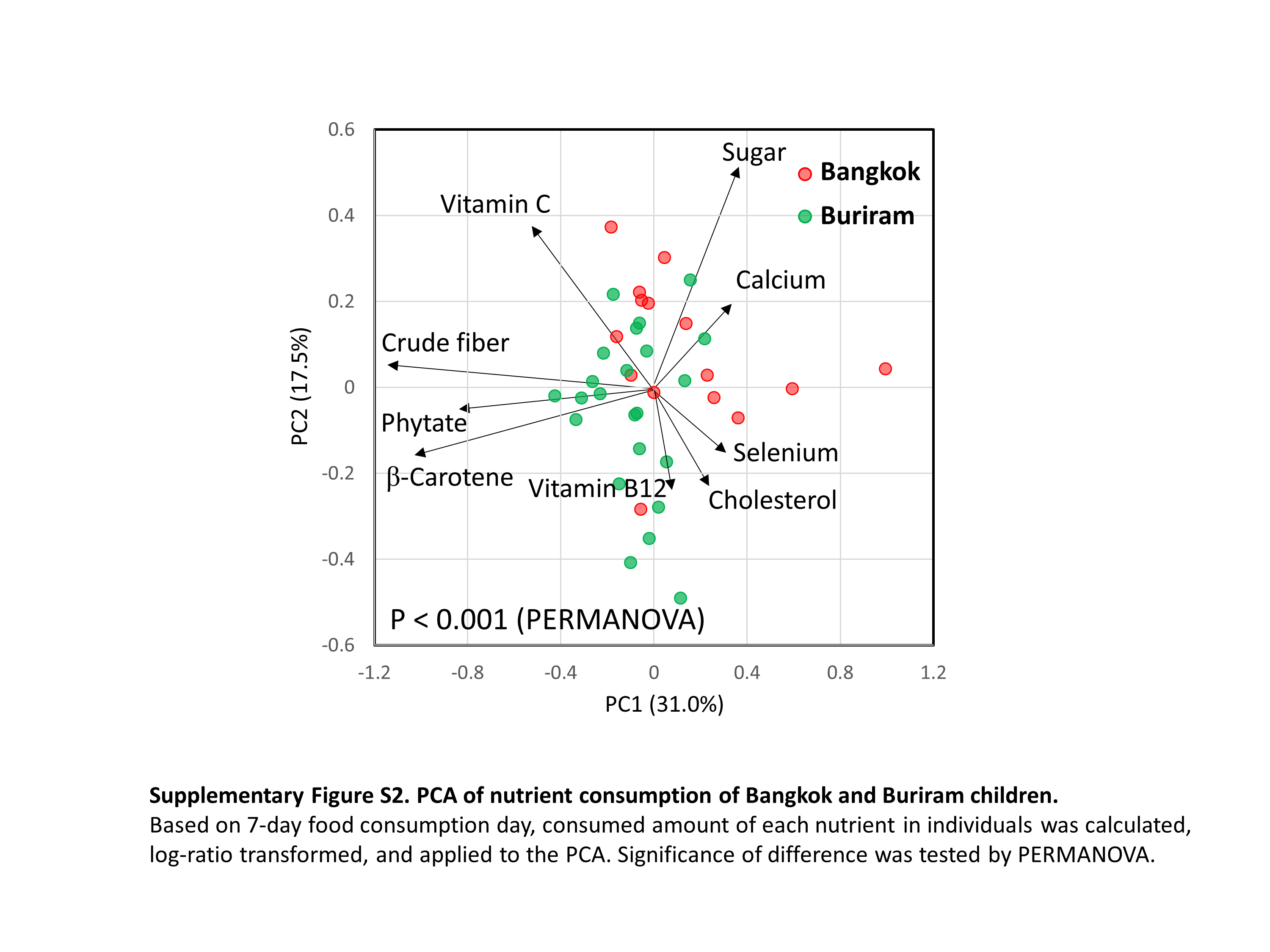

Supplement: Supplementary file 9 [file Image_2.TIF]

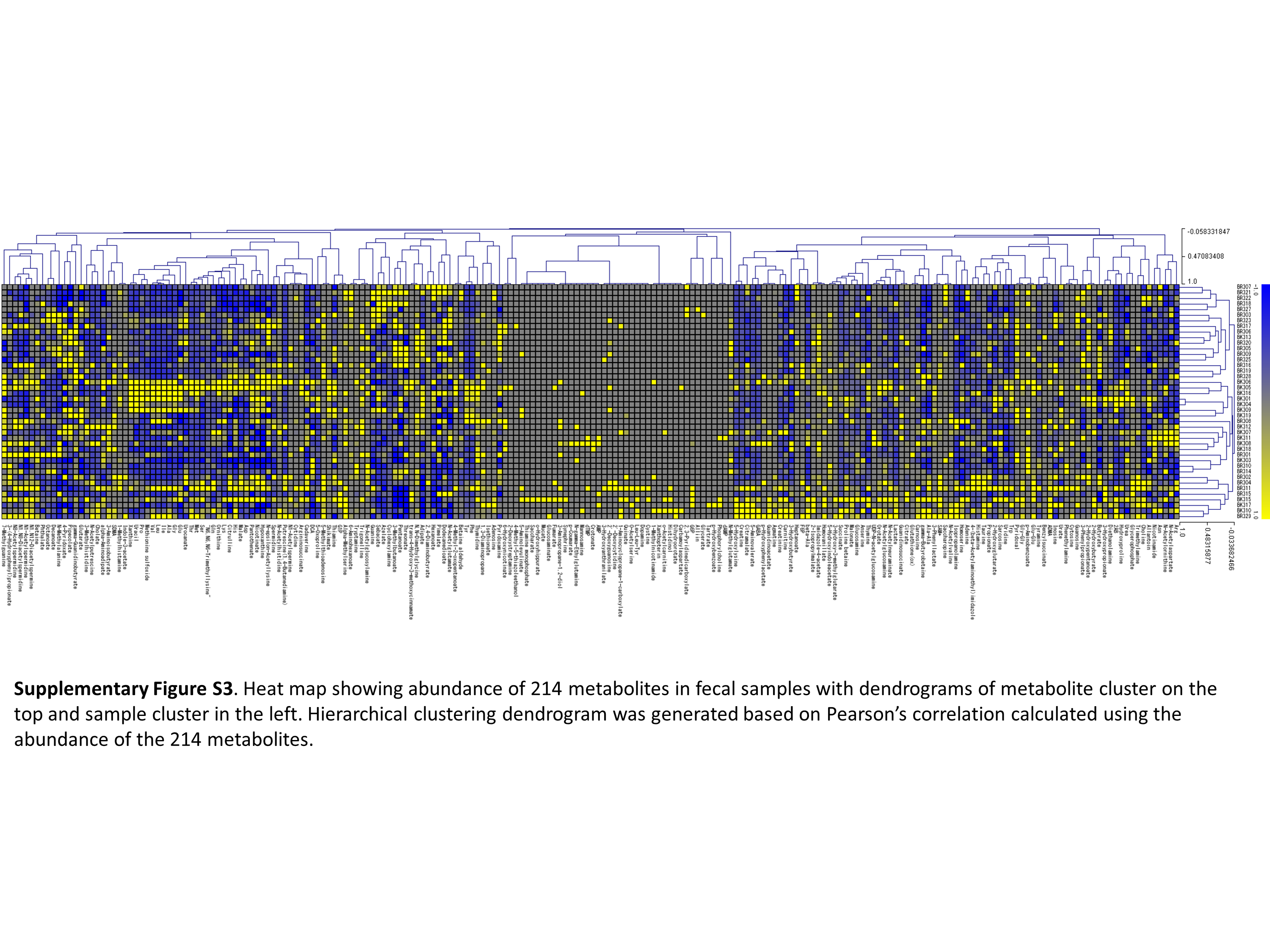

Supplement: Supplementary file 10 [file Image_3.TIF]

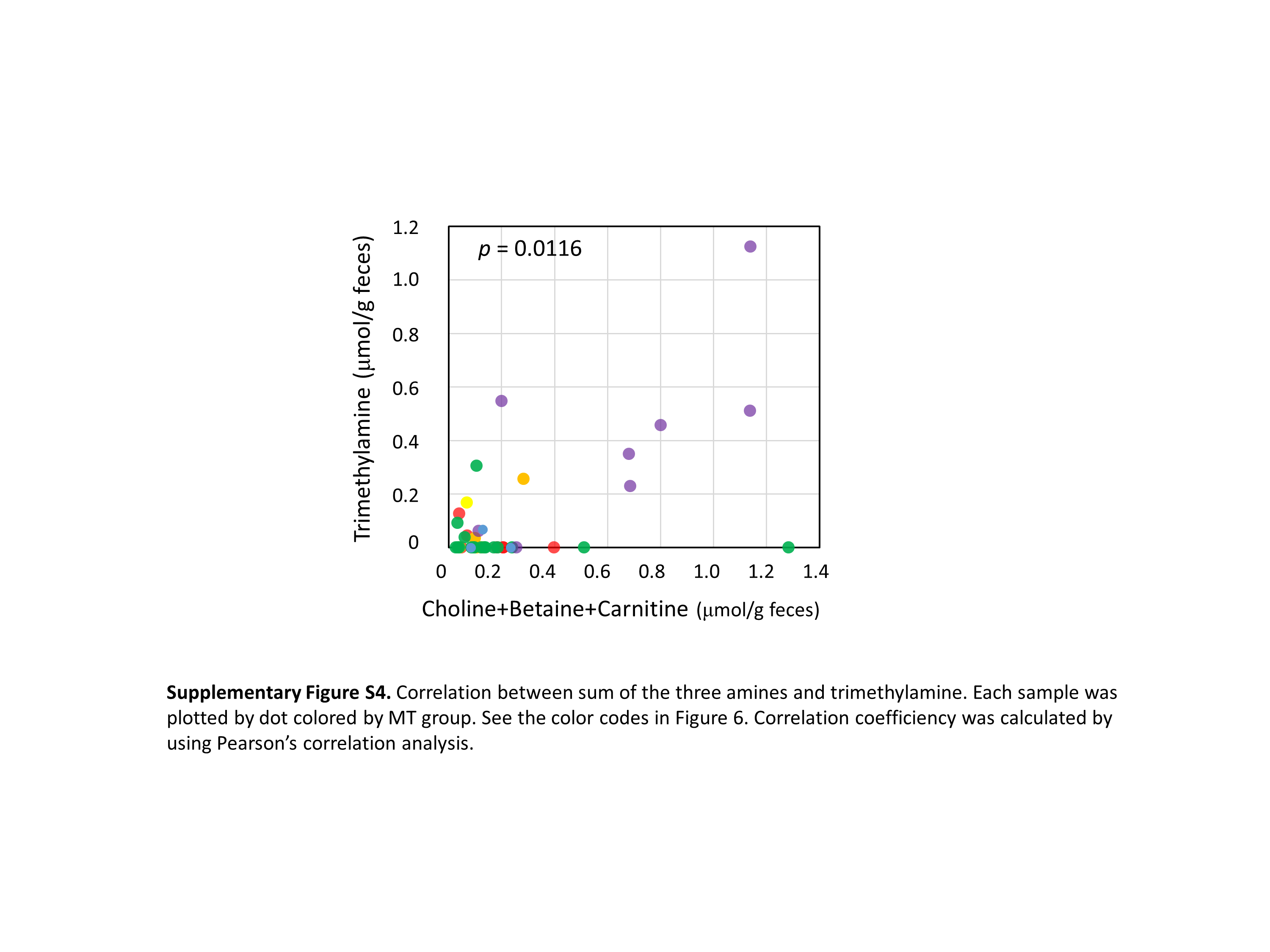

Supplement: Supplementary file 11 [file Image_4.TIF]

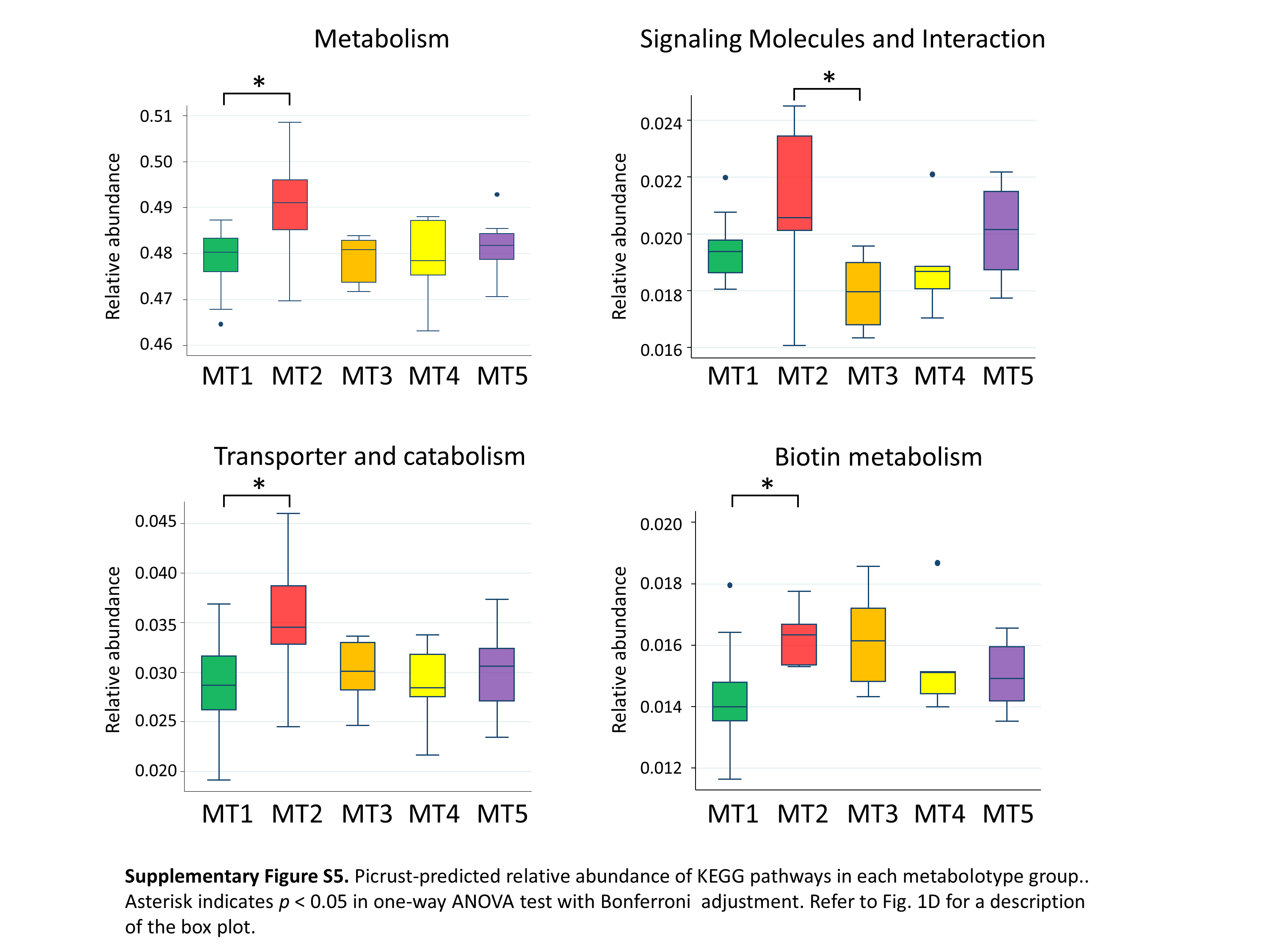

Supplement: Supplementary file 12 [file Image_5.TIF]
